# Supplementary material for: Regular testing of asymptomatic healthcare workers identifies cost-efficient SARS-CoV-2 preventive measures
Source: PLoS One. 2021 Nov 5;16(11):e0258700. doi: 10.1371/journal.pone.0258700 (PMC8570514; doi:10.1371/journal.pone.0258700)
Supplement: S1 File — Pseudocode with the stochastic simulation algorithm. (ZIP) [file pone.0258700.s001.zip › S1_File.pdf]

S1 File

This supplementary file is devoted to explain the simulation algorithm employed in the manuscript.

1. Set the initial condition for $(S_C, E_C, I_C, H_C, R_C, D_C) = (N, 0, 1, 0, 0, 0)$ at time $t = -8$ and integrate the ODE system 1-6 until day 15. Note that March 1st 2020 corresponds to $t = 0$.

2. set $(S1, E1, A1, I1, Q1, R1) = (157, 0, 0, 0, 0, 0)$,
   $(S2, E2, A2, I2, Q2, R2) = (157, 0, 0, 0, 0, 0)$, and start the stochastic simulation.

   (a) Select reaction r_i and Δt according to the Gillespie algorithm. That is, Let p_1, p_2 two random numbers from a uniform distribution between 0 and 1. We select r_i as the smallest i that satisfies

   $$\sum_j^i W_j(x) > p_1 \sum_j W_j(x)$$

   and

   $$\Delta t = \frac{1}{\sum W_j(x)} \log\left(\frac{1}{p_2}\right)$$

   (b) update $(S1, E1, A1, I1, Q1, R1)$, $(S2, E2, A2, I2, Q2, R2)$ and t accordingly.

   (c) integrate system of ODE 1-6 for a time step of Δt.

   (d) If $t \in (15, 64)$

      i. if `floor(t/7)`$\%2 = 0$, then $\beta_2 = \gamma_2 = 0$, $\beta_1 = 1.5\beta, \gamma_1 = 1.5\gamma$.

      ii. if `floor(t/7)`$\%2 = 1$, then $\beta_1 = \gamma_1 = 0$, $\beta_2 = 1.5\beta$, $\gamma_2 = 1.5\gamma$.

      iii. if `floor(t)` $\%\ 7 = 1$ AND `TestDay=1` AND $t > 22$, we test

      - TestDay=0
      - Set Tests=E1
      - for (i = 0, i < Tests, i=i+1)
        if `random(0,1)` $< \epsilon_3\times$ Fraction of workers tested, then
        Q1=Q1+1
        E1=E1-1
      - Set Tests=A1
      - for (i = 0, i < Tests, i=i+1)
        if `random(0,1)` $< \epsilon_3\times$ Fraction of workers tested, then
        Q1=Q1+1
        A1=A1-1
      - Set Tests=E2
      - for (i = 0, i < Tests, i=i+1)
        if `random(0,1)` $< \epsilon_3\times$ Fraction of workers tested, then
        Q2=Q2+1
        E2=E2-1
      - Set Tests=A2

- for (i = 0, i < Tests, i=i+1)
  if `random(0,1)` $< \epsilon_3 \times$ Fraction of workers tested, then
  Q2=Q2+1
  A2=A2-1

iv. if `floor(t)` % 7 $\neq$ 1: TestDay=1

(e) If $t > 64$

- S1=S1+S2, S2=0
- E1=E1+E2, E2=0
- A1=A1+A2, A2=0
- I1=I1+I2, I2=0
- Q1=Q1+Q2, Q2=0
- R1=R1+R2, R2=0
- $\beta_1 = \beta, \gamma_1 = \gamma$
- if `floor(t)` % 7 = 1 AND `TestDay=1`, we test
  - TestDay=0
  - Tests=E1
  - for (i = 0, i < Tests, i=i+1)
    if `random(0,1)` $< \epsilon_3 \times$ Fraction of workers tested, then
    Q1=Q1+1
    E1=E1-1
  - Tests=A1
  - for (i = 0, i < Tests, i=i+1)
    if `random(0,1)` $< \epsilon_3 \times$ Fraction of workers tested, then
    Q1=Q1+1
    A1=A1-1
- if `floor(t)` % 7 $\neq$ 1: TestDay=1

3. Go to (a) or END if $t > 91$.

For the second wave, the algorithm reads

1. Set the initial condition for $(S_C, E_C, I_C, H_C, R_C)$ their value at time $t = 91$ and the time t at 91.
2. set $(S, E, A, I, Q, R) = (306, 0, 0, 0, 0, 8)$, and start the stochastic simulation.

   (a) Select reaction r_i and Δt according to Gillespie algorithm

   (b) update (S, E, A, I, Q, R) and t accordingly.

   (c) integrate system of ODE 1-6 for a time step of Δt.

   (d) if `floor(t)` % 7 = 1 AND `TestDay=1`, we test

   - TestDay=0
   - Tests=E
   - for (i = 0, i < Tests, i=i+1)
     if `random(0,1)` $< \epsilon_3 \times$ Fraction of workers tested
     Q=Q+1
     E=E-1
   - Tests=A

- for (i = 0, i < Tests, i=i+1)
  if `random(0,1)` $< \epsilon_3 \times$ Fraction of workers tested
  Q=Q+1
  A=A-1

(e) if `floor(t)` % $7 \neq 1$, then TestDay=1

3. Go to (a) or END if $t > 365$.
