# Supplementary figures and images for: Regular testing of asymptomatic healthcare workers identifies cost-efficient SARS-CoV-2 preventive measures
Source: PLoS One. 2021 Nov 5;16(11):e0258700. doi: 10.1371/journal.pone.0258700 (PMC8570514; doi:10.1371/journal.pone.0258700)

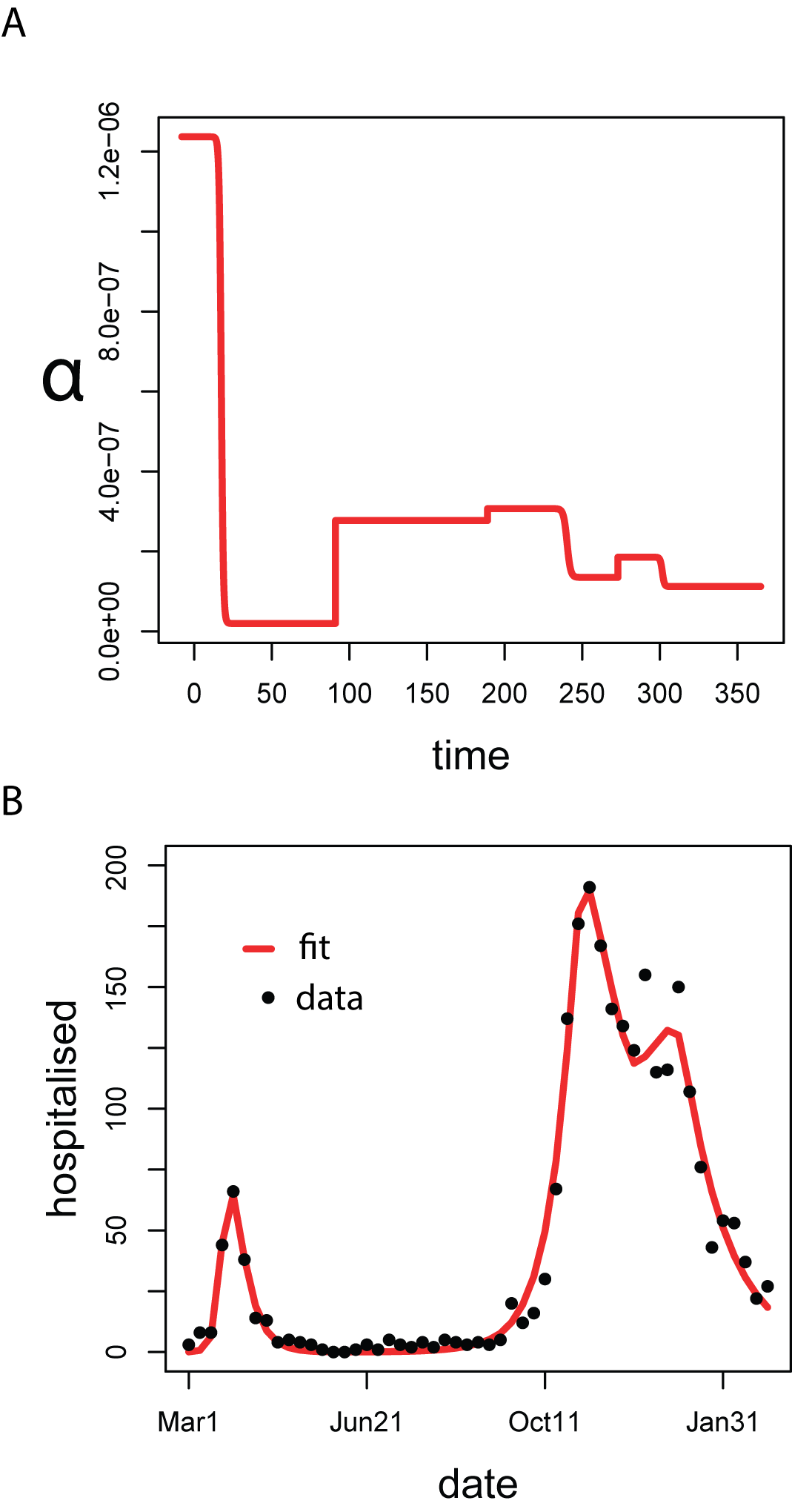

Supplement: S1 Fig — Figure legend: (A) value of α(t) during the simulation period. (b) sum of the last 7 days of HC(t) (red) and sum of the last 7 days of reported hospitalizations (black points). (PNG) [file pone.0258700.s003.png]

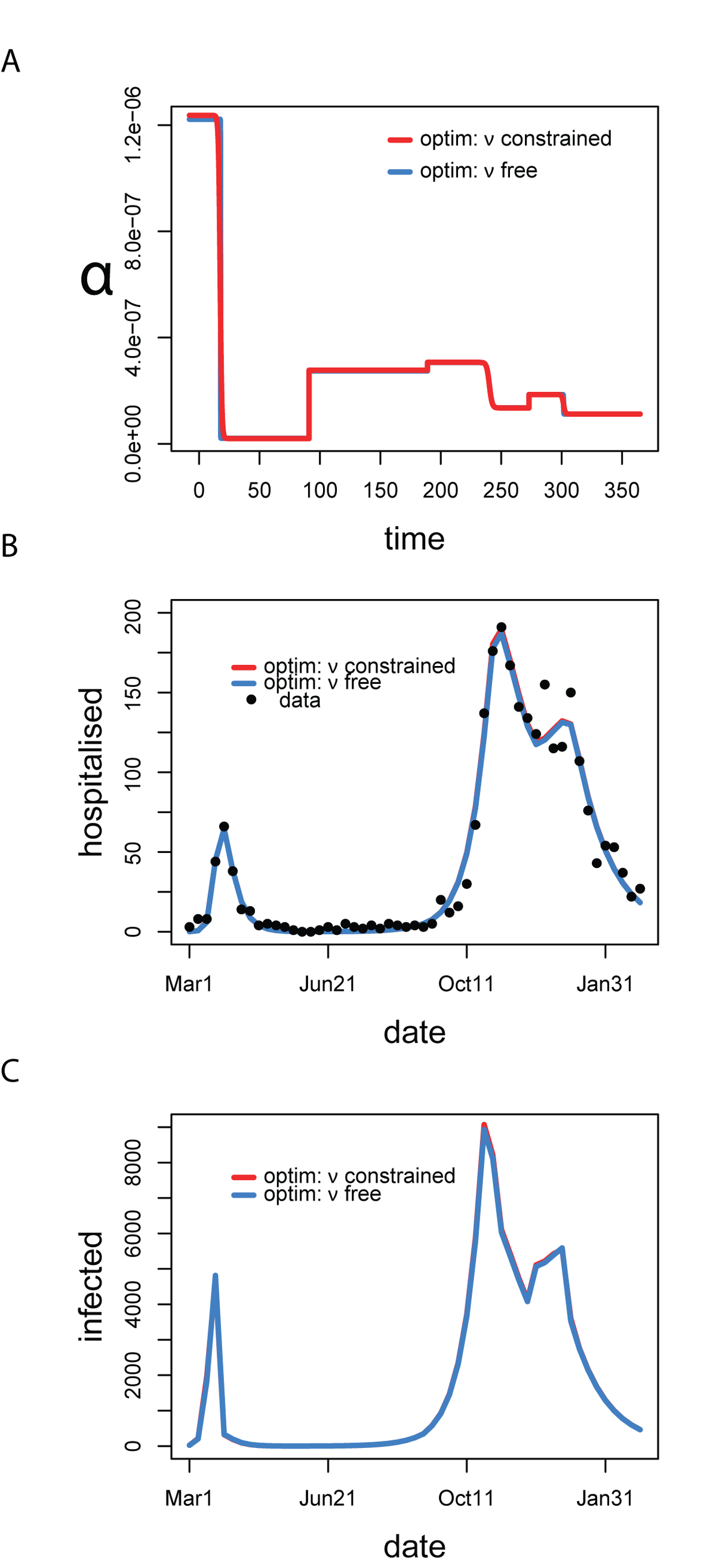

Supplement: S2 Fig — Figure legend: Effect of the constraints on ν on the fit (a) α(t) (b) Number of hospitalizations during the last 7 days (c) Number of infections during the last 7 days. (PNG) [file pone.0258700.s004.png]
