# Supplementary material for: Regular testing of asymptomatic healthcare workers identifies cost-efficient SARS-CoV-2 preventive measures
Source: PLoS One. 2021 Nov 5;16(11):e0258700. doi: 10.1371/journal.pone.0258700 (PMC8570514; doi:10.1371/journal.pone.0258700)

We thank the reviewers for their comments and suggestions that we believe have improved the quality of our manuscript significantly. All the issues raised by the reviewers have been addressed as shown below.

Editor

1. Please ensure that your manuscript meets PLOS ONE's style requirements, including those for file naming. The PLOS ONE style templates can be found at https://journals.plos.org/plosone/s/file?id=wjVg/PLOSOne_formatting_sample_main_body.pdf and https://journals.plos.org/plosone/s/file?id=ba62/PLOSOne_formatting_sample_title_authors_affiliations.pdf

The manuscript now meets the requirements of PLOS ONE as indicated.

2. Please provide additional details regarding participant consent. In the Methods section, please ensure that you have specified (1) whether consent was informed and (2) what type you obtained (for instance, written or verbal). If your study included minors, state whether you obtained consent from parents or guardians. If the need for consent was waived by the ethics committee, please include this information.

Informed and written consents was obtained from all participants according to the requirements of the local ethical committee.

3. Please note that PLOS ONE has specific guidelines on code sharing for submissions in which author-generated code underpins the findings in the manuscript. In these cases, all author-generated code must be made available without restrictions upon publication of the work. Please review our guidelines at https://journals.plos.org/plosone/s/materials-and-software-sharing#loc-sharing-code and ensure that your code is shared in a way that follows best practice and facilitates reproducibility and reuse.

Supplementary materials contain a pseudocode of the stochastic simulation algorithm. The code for the optimization and the code to perform the stochastic simulations will be included on github upon publication.

4. We note that the grant information you provided in the 'Funding Information' and 'Financial Disclosure' sections do not match.
When you resubmit, please ensure that you provide the correct grant numbers for the awards you received for your study in the 'Funding Information' section.

Please see reply to point 5 just below.

5. Thank you for stating the following in the Acknowledgments Section of your manuscript:
"The study was supported by the Swiss National Science Foundation with the grants 196059 (G. B., D. S.-T.) and 196641 (S. G.-V.) and by the Bern Center for Precision Medicine (G. B., D. S.-T.). "

We note that you have provided funding information that is not currently declared in your Funding Statement. However, funding information should not appear in the Acknowledgments section or other areas of your manuscript. We will only publish funding information present in the Funding Statement section of the online submission form. Please remove any funding-related text from the manuscript and let us know how you would like to update your Funding Statement. Currently, your Funding Statement reads as follows:
"The study was supported by the Swiss National Science Foundation with the grants 196059 (G. B., D. S.-T.) and 196641 (S. G.-V.) and by the Bern Center for Precision Medicine (G. B., D. S.-T.).
The funders had no role in study design, data collection and analysis, decision to publish, or preparation of the manuscript."
Please include your amended statements within your cover letter; we will change the online submission form on your behalf.
We agree with the Funding Statement as proposed:
"The study was supported by the Swiss National Science Foundation with the grants 196059 (G. B., D. S.-T.) and 196641 (S. G.-V.) and by the Bern Center for Precision Medicine (G. B., D. S.-T.). The funders had no role in study design, data collection and analysis, decision to publish, or preparation of the manuscript."

6. One of the noted authors is a group or consortium UVCM-COVID researchers. In addition to naming the author group, please list the individual authors and affiliations within this group in the acknowledgments section of your manuscript. Please also indicate clearly a lead author for this group along with a contact email address.
The affiliations of the authors in the consortium and a lead author have been included in the acknowledgement section.

Reviewer #1: Review Comments

1. In the abstract section, please add appropriate key words like testing, SARS COV 2and asymptomatic

The following keywords have been added to the abstract: **SARS-CoV-2, Asymptomatic, Healthcare Workers, Stochastic Modelling, Testing Strategies.**

2. Add the study design you have used in the abstract section

We have performed a cohort study that included asymptomatic healthcare workers of the Department for Visceral Surgery and Medicine of the Bern University Hospital. This information has now been included in the abstract and the methods section.

3. Well-articulated writing style in introduction part but didn't show the gap why the importance of conducting this study for health workers and patients apart from entire world. Please try to state the importance of conducting this research in the revised manuscript

In the introduction we have described that "However, applying some of these measures among healthcare workers is challenging, as they need to work in close contact with infected patients and with their coworkers, and as the possibilities for home-office options are limited. Therefore, the healthcare workforce could be exposed to an increased risk of infection transmission than the general population and devising protective strategies for them remains a crucial task."

In the discussion we have now further strengthened this aspect in the first paragraph to show the relevance of our models for testing in healthcare workers.

4. In the method part, better to include sampling procedures

The sampling procedures are now described in detail in the third subsection of the Materials and Methods.

5. Have you checked the confounding factors during testing? Please stated it in the revised manuscript whether you consider confounding variable or not

For our study, we did not consider confounding factors during the testing. Mainly due to the small sample size, all the workers are assumed to be the same. This has been specified in the main text.

6. Write strengths and limitation of the study in the revised manuscript

We have extended the discussion section to indicate the strengths and, the limitations of, our study. In particular, that we have a unique set of data investigating healthcare workers from the onset of a pandemic. The study includes mechanistic mathematical models that account simultaneous differences in dynamics between the city and in a hospital. This model can be adapted to other scenarios with a different number of cases.

The limitations are that the dataset accounts for only one department and cannot be extrapolated to cases where hospitals were saturated.

7. Include Conclusion section following discussion part and place recommendation for future investigators

We have adapted to Discussion section to finish with a Conclusion section, where we have also indicate possible future directions.

General comments

-The research is novel and will be an input for scientific community after publication

- The authors followed all scientific procedures to conduct the study except some minor comments raised in the above comments

Thank you for these positive comments.

Reviewer #2: Summary:
The authors aim to investigate a set of coupled models to explain the spread of SARS-CoV-2 not only in the Swiss Canton of Bern but also within the local hospital environment.

To do so they present two coupled models. The first is a mean-field compartmental infectious disease model to describe the dynamics of the virus within the surrounding community. The second is a stochastic model (via Gillespie simulations of related ODEs) of the viral dynamics within the healthcare workers.

To parameterize these models, the cumulative infections within Bern were used. Additionally PCR data from voluntary swabs of healthcare workers were used to parameterize the Gillespie model.

Remarks:
1. Numerous times the authors refer to the cycle threshold (Ct), though this quantity is never defined nor explained anywhere in the manuscript (in fact, I do not believe the words "Cycle Threshold" appear anywhere within the manuscript). This quantity should be defined/explained and its importance emphasized within the manuscript. (Perhaps in the RNA and RT-qPCR section). Notably some intuition should be given for the importance of the size / relative ordering of these values. What does a mean Ct of 20 intuitively mean, for the symptomatic group, compared to a Ct of 35 (Fig 1D), for example.
For the PCR we selected a genetic region of interest (specific for the virus), and a primer to target it. In each cycle, a polymerase will capture the primers bound to the RNA present in the sample and duplicate them. After a certain amounts of duplications, the presence of RNA is detected giving a cycle threshold value (ct-value). Thus, the viral load is higher in the test with a ct-value of 20 (cycles) compared to 32 (cycles).
We have explained what a Cycle threshold value is at the end of the subsection RNA extraction and RT-qPCR in the methods section, and how to interpret it.

2. The presentation of the pseudo-code on pages 7-9 should be re-considered. In particular, the pseudocode switches between being quite high-level (Select reactions according to Gillespie's algorithm; update the states accordingly) and being quite low-level (explicit for loops in C-like syntax, etc.) In particular, the authors employ a flag variable 'Bol' with a very un-informative name. The flag, presumably, is included so as to ensure that there isn't simulated "double testing" if the Delta t chosen by Gillespie's algorithm is smaller than one day. For some reason, this flag seems to be improperly employed in the t>64 case of the algorithm. (Bol is never reset to 0 suggesting a possibility of this spurious "double testing"). The authors should ensure that this error is only present in the presentation of the algorithm and not in the actual simulations.
The authors should decide if they want to provide a high-level overview here, or a low-level implementation. If the former, then point 2d) and 2e)could be re-written to be more human-readable. If the latter, then there certainly shouldn't be any implementation glitches present within the summary of the algorithm.
Finally, a minor critique, the ending condition for the algorithm is never specified.

Presumably after some time point was reached? Should step 3 read: "Go to 2(a) or END if t=365"?
We agree with the reviewer that pages 7-9 are confusing. We have rewritten that section to show a high-level overview. The low-level pseudocode is included in the supplementary materials.

3. The quantity f_p is introduced (line 171 on page 6) and then never used again or explained. (In the implementation of the algorithm, the phrase "Fraction of workers tested" is employed instead, presumably this refers to f_p). It is also unclear to me *what* f_p is. Is f_p a time dependent parameter? f_p is not present in the parameter value table, perhaps f_p is calculated on a per-day basis from the PCR data, in any case this should be made clear.
We apologize for this mistake in the notation. We have added f_p to table 2 and now it is described and used as the other parameters.
We have extended the explanation of f_p in the methods, where we state that on average, based on our data, 65% were tested before day 91 (first wave) and 45% after. These averages were computed as number of total tests/number of total participants.

4. A few typographical issues:
- In the text on lines 180-181, the authors indicate that tau, pi, nu, and kappa are fit via minimizing this cost function. However the cost function as written is only a function of kappa, nu, and pi (notably: not a function of tau, is there a reason for this? Or is there just a typo in line 180 or in the definition of G).
- the integral between lines 181 and 182 is missing the variable of integration (presumably time).
These and all the other typos have been corrected.

- Lines 51-52 include the sentence "We fitted the model to the recorded number of hospitalized individuals outside the hospital". Presumably the authors mean "infected individuals outside the hospital".
We apologize for this error. We meant the recorded number of hospitalized individuals in the city, which was the quantity used to estimate the parameters of the model. This point has been corrected.

- On line 164 the authors say beta_1(t) = 1.5\beta, \gamma_1(t)=\gamma. and then one line 165, when the desynchronized cohorts switch, they say beta_2(t)=1.5\beta, \gamma_2(t)=1.5\gamma. I assume there is a typo in the definition of \gamma_1(t) on this interval (probably meant \gamma_1(t)=1.5\gamma). If this isn't a typo, this discrepancy should be explained. Also, on lines 216 and 217 they define \gamma_1=\gamma and \gamma_2=\gamma and not 1.5\gamma, in contradiction with definitions of \gamma_i(t) earlier and in contradiction with line 166 that states "We assume an increase of 50% in beta and gamma..."
- Perhaps a nitpicky critique, but it would be good around lines 155-167 to discuss WHEN the bare values of \gamma and \beta are actually used. The definition of \beta_i switching

between 1.5\beta and 0 in lines 155-167 naturally raises the question: why not just redefine \beta as this 50% increase of the nominal value? This isn't answered until a full two-pages later in line 248 where it becomes clear that the bare value of \beta is used within the simulations when desynchronization is not considered.
- There are overbars on some quantities in equations (9) and (10). Are these just typographical errors or is this meant to indicate something else? Notably these bars are absent in the corresponding terms in Table 1.
All these typos have been corrected

5. Perhaps I am missing something, but I fundamentally do not understand the point being made in lines 283-285. While I understand that without this artificial reaction there may be situations where large values of delta t are chosen by Gillespie's algorithm, I fail to understand how this relates to errors in the solution of the ODE system. My understanding of the algorithm is as follows:
- The city ODE model is integrated for 8 days. During this time the Gillespie model is not being used.
- Then the Gillespie model is initialized and chooses a reaction and a delta t. The Gillespie state vectors are updated accordingly.
- Then the city ODE model is integrated for delta t days.
- (Then testing updates, desynch turning on and off, etc.)
I assume that the large delta t values that "lead to errors in the solution of the ODE system" that the authors refer to would be introduced at this third step of my summary. I just fail to see why that is necessarily the case. delta t, the step size for the Gillespie simulation, is not necessarily the step size for the ODE solver. It is just the final time of the time-mesh used by the ODE solver. Why not define a maximum step size, h_t say, that is sufficiently small (i.e. h_t=0.01 or smaller) and integrate the city ODE over the time mesh [t_i, t_i + h_t, t_i + 2 h_t, ..., t_i + delta t]. You then will not be sacrificing accuracy in the integration of the ODE system while not introducing an artificial reaction in the Gillespie simulation.
Partially the reason this concerns me is that with the artificial reaction I am not convinced that a Gillespie simulation is actually being employed. i.e. It almost reads as just a Monte-Carlo step with a completely spurious temporal update.
We have added an explanation of the empty reaction. As the reviewer suggested, it has no impact on the ODE. But it can create problems when coupling a system of ODEs and a stochastic system if the step sizes are large. Namely, when \alpha(t) is small and all workers are susceptible or recovered, the Gillespie algorithm would select a large step size. However, \alpha can increase substantially during that large period, leading to possible errors.

Since the stochastic system is coupled to an ODE system, the simulation method differs slightly from the traditional Gillespie algorithm, but the step size and the state change reactions are selected in the same way.

6. It is not clear to me why alpha(t) switches form throughout the simulation. It is this Holling Type-2 function during waves 1 and 2 and during the winter after wave 2, but a

constant function between waves 1 and 2. Is there any explanation as to why this choice was made? What happens if between waves it is treated as having the same functional form? Additionally, it would be good to see a plot of alpha(t) for all time being considered (perhaps in the SI, at the authors' discretion).
During waves 1 and 2 there are non-therapeutic interventions that can match the days where alpha changes. However, during summer, we assume alpha(t) = pi/N. Representing a scenario were the pandemic is controlled. This assumption is equivalent to kappa=1.

For wave 2 we change the alpha back to the Holling Type-II function, where the pandemic became uncontrolled, e.g. contract-tracing was overwhelmed, and new non-therapeutic interventions were needed. We could have tried to generate an alpha that fits the summer and wave 2 together, but due to the low number of cases and hospitalization in the summer period, we did not consider it as necessary.

7. The fitting/optimization process is not discussed in nearly enough detail. Is it just a brute force method over the lattice of values presented in the "Model Fitting - City" section? Is some other optimization algorithm being employed instead?
Initially we used a brute force algorithm. However, the reviewer point made us consider the option of trying a more sophisticated algorithm, and the optimization has been repeated with the R function *optim* with the parameter *method*="*L-BFGS-B*". None of the results of the paper were effected, but we identified some small quantitative differences and we considered that it was necessary to modify.
To avoid local minimums, we have repeated the simulations with 1000 initial conditions, in the parameter region
-pi=[0,2], kappa=[0,0.15], nu=[0,1.5], and tau=[15,24] for wave 1.
-pi=[0,1] for wave summer.
-pi=[0,0.7], kappa=[0,1], nu=[0,1.5], and tau=[230,246] for wave 2.
-pi=[0,0.5], kappa=[0,1], nu=[0,1.5], and tau=[295, 305] for wave winter

The results of the optimization are
-tau=17.4352, pi= 1.2801, kappa=0.016, and nu=1.5$ for wave 1.
-pi= 0.2869 for wave summer.
-tau=240.2609, pi= 0.3177, kappa=0.44, and nu=0.8934, for wave 2
-tau = 301.6273, pi = 0.1919, kap = 0.6047, and nu = 1.5 for wave winter.
As expected, Alpha did not change substantially with the brute force algorithm or the *optim* R function.

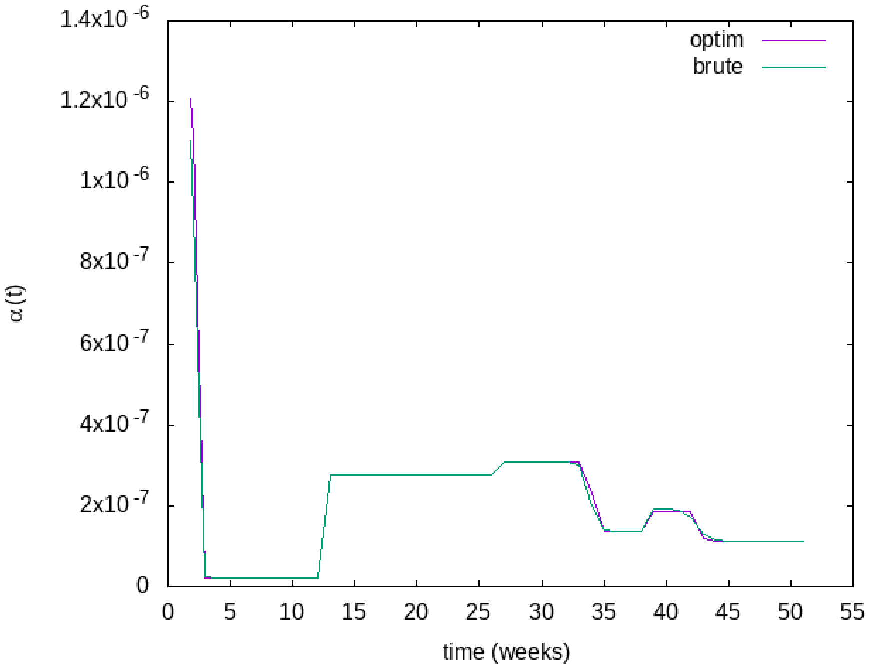

*Caption: Changes of a*lpha in time (green) brute force, (purple) *optim*.

Nor did the number of cases

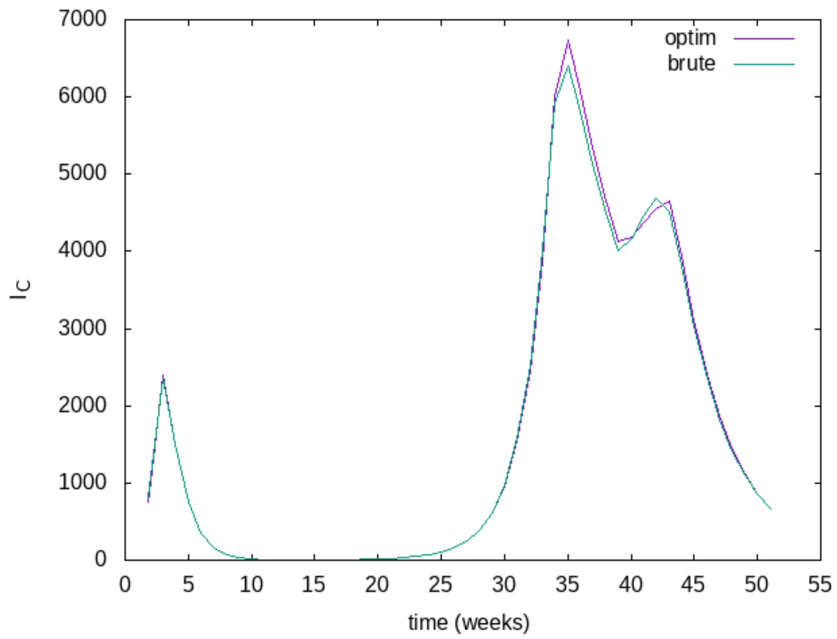

Caption: Number of infected citizens (green) brute force, (purple) optim.

Nor did the fit for the hospitalized

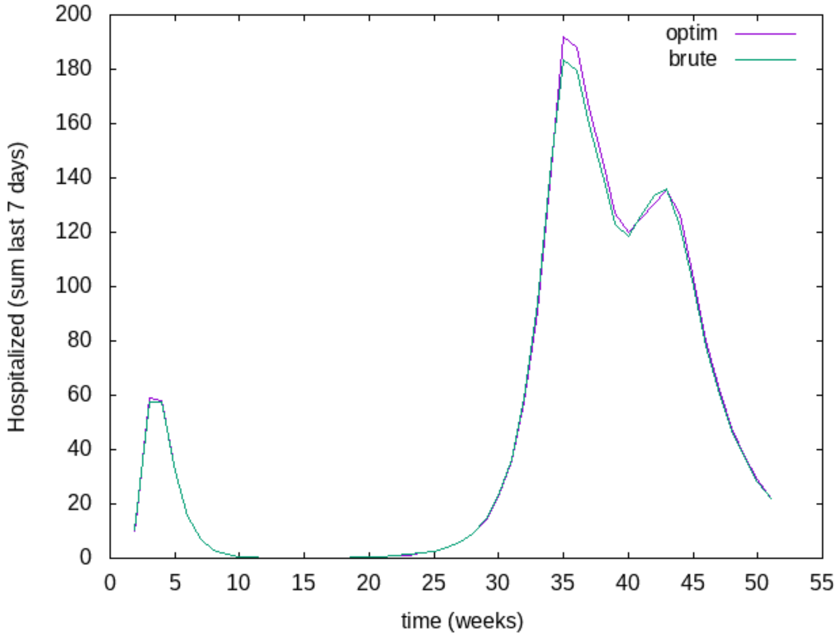

Caption: Hospitalizations of the last 7 days (green) brute force, (purple) optim.

However, for the first wave and the winter, the minimum was found at the edge, nu=1.5. We decided to restrict the value of nu to 1.5 or below, because we did not want to assume instantaneous effects in the non-therapeutic interventions. And for large values of nu it would produce a faster change in alpha that the temporal resolution of the data. This has been clarified in the text. Moreover, in Supplementary Figure 2 we show the different fit

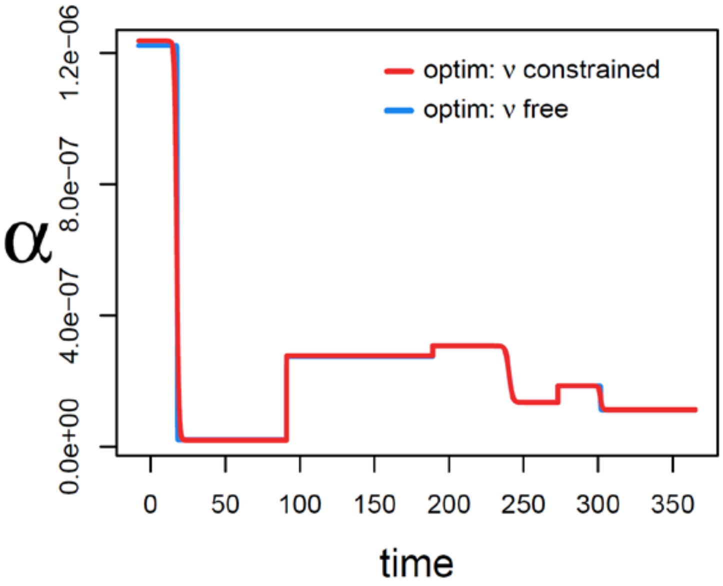

As expected, Figure 2 does not change regardless of the optimization method used. For example, Panel 2C now looks

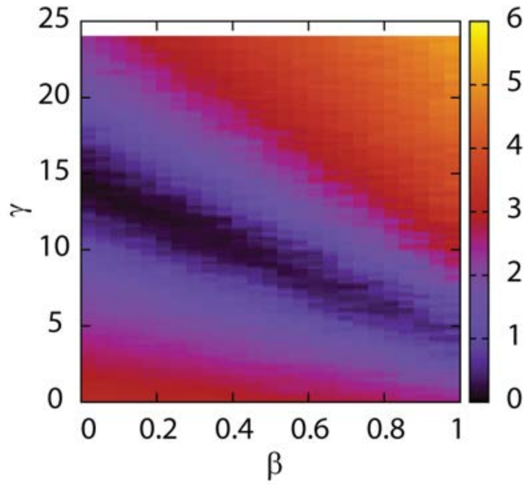

However, the better optimization algorithm had some impact on tau and nu. For this reason, we decided to update all the figures with the new values.

8. The authors (line 202-205) discuss how in actuality testing was performed on the in-hospital cohort only (on a voluntary basis) and not on the home-office cohort. They then simulate testing on both cohorts "for simplicity". I do not find this to be an acceptable assumption (though the authors may disagree with evidence to the contrary!). Simulating testing only once a week instead of twice a week is, admittedly, unlikely to change terribly much dynamically (and hence seems to be a reasonable assumption, though I still think it would be incredibly simple to simulate this testing twice a week instead, but that is of little consequence), however it is the other assumption here that concerns me the most: why not simulate tests on only one of the cohorts? This seems like an incredibly simple addition to add to the code, as presented, (something like 'if beta_2==0, then do the tests on E1 and A1, else do the tests on E2 and A2').
Also: why simulate these tests on *everyone*? Presumably the authors know how many people were tested within a given week and could include that relatively simply here?

During the desynchronisation, workers in the hospital were tested Monday and Thursday, while workers doing home office were not tested. Our assumption of simulating tests performed on the two groups once a week, is equivalent to assuming that the second testing was delayed to Monday. This has been clarified in the text. We agree, however, with the data we have that testing in home-office would be of importance as a significant fraction of healthcare workers seem to become infected at home. This, however, was not foreseen and therefore not performed in our testing pipeline.

As the reviewer suggested, we have repeated the simulations without this assumption. We modified our code to perform tests twice a week only on the group that is working at the hospital. Below we represent the fit of data, shown in Figure 2C, since the differences are minimal, we prefer to keep the simplified simulation to not include extra details in the algorithm.

The Figure 2C with testing Monday and Thursday during the desynchronization is show below.

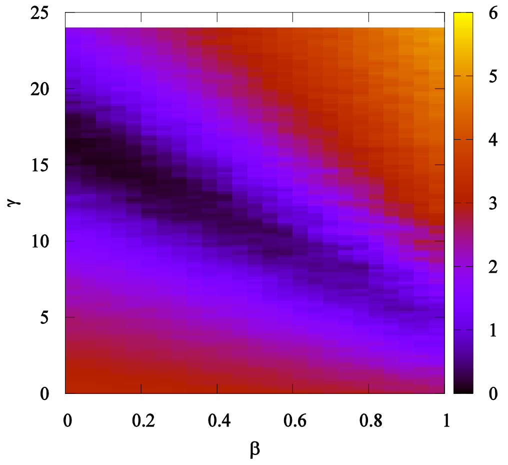

Regarding the workers tested. We have clarified that based on our data, an average of 65% of workers came to get tested during the first wave, and 45% afterwards. This has been clarified in method section where the f_p (see Reviewer 2 comment 3) is defined.

Based on Our data, an average of 65% came to become tested during the first wave, and 45% during the second.

These issues have been clarified in method section.

9. The Epi Data on lines 62-65 should be clarified. Are these daily time series or weekly time series? Are these cumulative hospitalizations, deaths, and cases or are they only new hospitalizations, deaths, and cases?
The Subsection Epidemiological data now includes a detailed description of the BAG data we used, and how it was employed in our simulations.

10. Paragraph on lines 286-289 should be rewritten for clarity. This is a particularly important part of the process and comprehension is harmed by the brevity. It is not clear to me what is happening in this paragraph; additional equations here would be an asset! You're redefining the cost function from Eqn. 15, so why not just present the actual functional form of the second cost function here and reduce ambiguity. What do you mean by "minimizing the function at times t=61 and 91"? Minimizing at time t=61 will give you one answer and at t=91 will give you a second, are you equally weighting these two options? Why not minimize the function over the whole time period, as is more commonly done? Or doing some kind of sum-of-squares of weekly minimization, as you did with the original cost function in lines 180-182?

We apologize for the confusing structure of that paragraph. We have rewritten it and now it includes the analogous equation to Eq. 15.

What do you mean by "minimizing the function at times t=61 and 91"? Minimizing at time t=61 will give you one answer and at t=91 will give you a second, are you equally weighting these two options? Why not minimize the function over the whole time period, as is more commonly done? Or doing some kind of sum-of-squares of weekly minimization, as you did with the original cost function in lines 180-182?

Because of the small number of positive tests, we decided to minimize the function using only the time points t=61 and t=91, because the first time point represents the end of the first peak, and the second time represents the beginning of the summer. When we include more time points (below, using the time points t=7,14,21, 28, 35, 42, 49, 56, 63, 70, and 77) we observe a similar qualitative behavior in the fit.

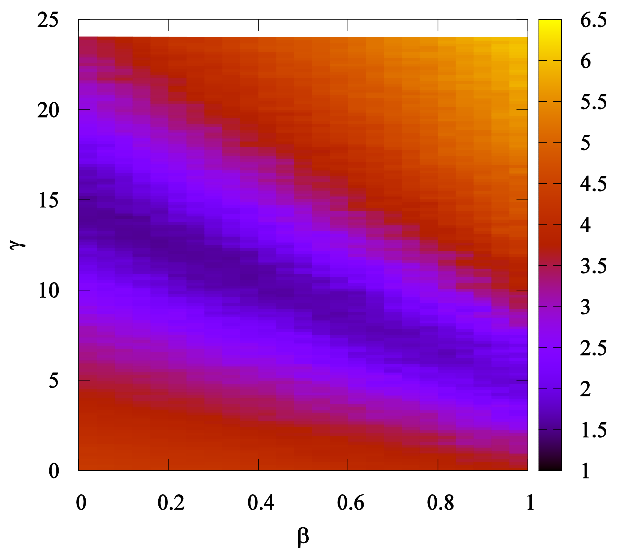

Supplement: Attachment — Submitted filename: responsetoreviewers.pdf [file pone.0258700.s006.pdf]
