# Supplementary material for: Regular testing of asymptomatic healthcare workers identifies cost-efficient SARS-CoV-2 preventive measures
Source: PLoS One. 2021 Nov 5;16(11):e0258700. doi: 10.1371/journal.pone.0258700 (PMC8570514; doi:10.1371/journal.pone.0258700)
Supplement: Attachment — Submitted filename: PLOS review.docx [file pone.0258700.s005.docx]

**Review Comments**

1. In the abstract section, please add appropriate key words like testing, SARS COV 2and asymptomatic
2. Add the study design you have used in the abstract section
3. Well-articulated writing style in introduction part but didn’t show the gap why the importance of conducting this study for health workers and patients apart from entire world. Please try to state the importance of conducting this research in the revised manuscript
4. In the method part, better to include sampling procedures
5. Have you checked the confounding factors during testing? Please stated it in the revised manuscript whether you consider confounding variable or not
6. Write strengths and limitation of the study in the revised manuscript
7. Include Conclusion section following discussion part and place recommendation for future investigators

**General comments**

**-**The research is novel and will be an input for scientific community after publication

- The authors followed all scientific procedures to conduct the study except some minor comments raised in the above comments
